# A modified algorithm with lipoprotein(a) added for diagnosis of familial hypercholesterolemia

**DOI:** 10.1002/clc.23251

**Published:** 2019-08-22

**Authors:** Di Sun, Ye‐Xuan Cao, Sha Li, Yuan‐Lin Guo, Na‐Qiong Wu, Ying Gao, Qiu‐Ting Dong, Geng Liu, Qian Dong, Jian‐Jun Li

**Affiliations:** ^1^ Division of Dyslipidemia, State Key Laboratory of Cardiovascular Disease Fu Wai Hospital, National Center for Cardiovascular Disease, Chinese Academy of Medical Sciences and Peking Union Medical College Beijing China

**Keywords:** diagnosis, DLCN, familial hypercholesterolemia, LDL‐C, lipoprotein (a)

## Abstract

**Background:**

Previous studies have observed that high level of lipoprotein (a) [Lp(a)] was common in the phenotypic familial hypercholesterolemia (FH) and may explain part of the clinical diagnosis of FH.

**Hypothesis:**

We aim to develop a modified model including Lp(a) and compare its diagnostic performance with Dutch Lipid Clinic Network (DLCN) criteria.

**Methods:**

Data of 10 449 individuals were utilized for the model establishment (7806 for derivation and 2643 for validation) from January 2011 to March 2018. The novel score model was modified on the basis of DLCN. Furthermore, 718 patients were screened for *LDLR*, *APOB*, and *PCSK9* gene mutations.

**Results:**

The novel modified model consisted of untreated low‐density lipoprotein cholesterol (LDL‐C) level, Lp(a), personal premature coronary heart disease (CHD), tendon xanthomas and family history of CHD and/or hypercholesterolemia. It has shown high discrimination (area under curve [AUC] 0.991, 95% confidence interval [CI[ 0.988‐0.994, *P* < .001) for distinguishing clinical FH from non‐FH diagnosed using DLCN. Furthermore, a concordance analysis was performed to compare the modified model with DLCN and it showed a good agreement with DLCN (*κ* = 0.765). External validation of the novel model also showed good accordance (*κ* = 0.700). Further genetic analysis showed that the agreements between the new model and mutation improved a little compared to that between DLCN and mutation.

**Conclusions:**

The novel modified model, including Lp(a), could provide new insights into FH diagnosis in Chinese population with more concerns on the patients with high level of Lp(a).

## 1 INTRODUCTION

Familial hypercholesterolemia (FH) has been recognized as an inherited disease with extremely elevated level of low‐density lipoprotein cholesterol (LDL‐C) and thus premature coronary heart disease (CHD).^1^ Patients with homozygous phenotype even could suffer cardiovascular morbidity and mortality in their childhood.^2^, ^3^ Despite of increasing awareness of FH, it has still been underdiagnosed and undertreated worldwide, partly attributing to the complexity and disunity of the current diagnostic criteria and underutilized genetic testing.^4^

Until now, Dutch Lipid Clinic Network (DLCN) criteria, Simon Broome Register (SBR), Make Early Diagnosis‐Prevent Early Death (MEDPED), criteria given by the 2015 American Heart Association scientific statement and other national diagnostic algorithms have been adopted worldwide.^5^, ^6^, ^7^, ^8^ However, there are several limitations in regard of their utilization in the clinical practice. First, the cut‐off value of LDL‐C may be only applicable to specific populations. Second, a comprehensive family history of dyslipidemia and/or CHD is usually unavailable or inaccurate.^9^ Third, the discordance between different clinical diagnostic criteria and heterogeneity of phenotype and genotype often confused the physicians,^10^ especially for those without their own diagnostic guidelines including China. Last but not least, the calculation is complex and could not promote diagnosis conveniently in the primary care.

With the deeper understanding of FH, more factors have been described responsible for the manifestations, among which lipoprotein (a) [Lp(a)] has received a lot of attention.^11^, ^12^ Previous studies have demonstrated significantly higher level of Lp(a) in patients with FH compared to the non‐FH and its independent role in the risk stratifications.^13^, ^14^ The biochemical measurement of Lp(a) may explain 5% to 20% prevalence of the suspected FH, especially for those with negative FH‐causing mutations.^15^ Thus, we try to propose a potential approach to integrate Lp(a) into the diagnosis and investigate its performance.

Several novel model tools have been developed to simplify and improve the diagnosis of FH.^16^, ^17^ Recently, Ruel et al have proposed a simplified Canadian definition for FH, which had a comparable performance with the existing criteria SBR and DLCN.^18^ Unfortunately, studies addressing this issue for Chinese population have been lacked. We aim to develop a modified model including Lp(a) to facilitate the diagnosis of FH in the clinical practice, which could be adapted to Chinese population well.

## 2 MATERIALS AND METHODS

### 2.1 Study population

Our study complied with the Declaration of Helsinki and was approved by the hospital's ethical review board (Fu Wai Hospital and National Center for Cardiovascular Diseases, Beijing, China). Informed written consents were obtained from all the participants.

From March 2011 to March 2018, we consecutively recruited the subjects who were referred for coronary angiography (CAG) in this study as previously described.^19^ Patients were excluded if they: (a) with secondary cause of dyslipidemia including severe thyroid, liver, and renal dysfunction; (b) without LDL‐C measurement; (c) without Lp(a) measurement. As a result, a total of 10 449 participants at the time of the analysis were enrolled.

The adult patients were diagnosed as definite or probable FH according to DLCN criteria with a score ≥ 6. Clinical data of each participant were collected by physicians and experienced nurses, including the prior lipid levels and use of lipid‐lowering medications, family and personal history of dyslipidemia and CHD as well as presence of tendon xanthoma and corneal arcus. For patients on lipid‐lowering medications and without available untreated lipid profiles, their untreated LDL‐C levels were adjusted using a correction factor depending on the type and potency of the lipid‐lowering drugs.^20^ Of the 10 449 patients, we further enrolled 718 patients with LDL‐C levels above 4.5 mmol/L for a genetic testing. Patients were mutation positive if they carried pathological mutations in *LDLR*, *APOB* and *PCSK9* genes as described in our previous studies.^19^

### 2.2 Biochemical examination

After an overnight fast, blood samples were collected from cubital veins for biochemical measurements as described in our previous publications.^21^ Serum total cholesterol (TC), triglyceride (TG), high‐density lipoprotein cholesterol (HDL‐C) and LDL‐C were determined using an enzymatic assay with automatic biochemistry analyzer (Hitachi 7150, Tokyo, Japan). The concentrations of apolipoprotein (apo B) were measured by a turbidimetric immunoassay. Lp(a) was determined by immunoturbidimetry method (LASAY Lp(a) auto, SHIMA Laboratories Co., Ltd.). The detection range was 0.5 to 100 mg/dL with a normal value of below 30 mg/dL.

### 2.3 Model establishment

We developed the novel diagnostic criteria including Lp(a) on the basis of DLCN. First, we redefined the cut‐off value of LDL‐C according to Chinese data considering of the ethnic differences. Data from a cohort of patients without lipid‐lowering therapy (LLT) were used to determine the 95th percentile for LDL‐C. Furthermore, we calculated the best cut‐off value of LDL‐C for predicting FH and the value was 4.68 mmol/L. considering the relatively lower level of lipid in Chinese population, we adjusted the score classification and gave no score to the patients with LDL‐C < 4.7 mmol/L. Second, Lp(a) level ≥ 22 mg/dL was given one point. Third, the premature CHD was still given two points, which was defined as male patients with on‐set CHD younger than 55 years old and female patients younger than 60 years old. Of note, the xanthomas were specially referred to tendon xanthomas. Other manifestations including xanthelasma do not have high specificity although with a higher prevalence. Last but not least, as an inherited disorder, the family history is important for assisting diagnosis. In the new modified score model, we still kept one point for the family history of CHD or hypercholesterolemia. Finally, when the total score was above 6, the patient was defined as FH as same as DLCN criteria.

### 2.4 Statistics

The statistical analysis was performed using SPSS version 21 software (SPSS Inc., Chicago, Illinois). Continuous variables were presented as mean ± standard deviation (SD) or median (Q1‐Q3 quartiles) according to their distributions. Otherwise, categorical variables were shown as number (percentage). The differences of clinical and biochemical parameters between two groups were assessed using Student's *t* test, Mann‐Whitney *U* test or χ² test appropriately. In the current analysis, the prior 75% samples of the study population according to the recruitment time were used as the derivation cohort, while the remaining 25% samples were used to validate the model as the validation cohort. Patients with a score < 6 using DLCN criteria were designated as negative cases for calculating sensitivity, specificity, positive predictive value, and negative predictive value. The Cohen kappa (*κ*) coefficient was applied to evaluate the agreement between the new FH definition, DLCN criteria and gene mutation. In detail, *κ* > 0.8 indicated excellent agreement, 0.6 to 0.8 indicated good agreement, 0.4 to 0.6 indicated moderate agreement, and < 0.4 indicated poor agreement.^22^ The discriminative power was further evaluated using the receiver operating characteristics (ROC) curve and the new modified score model was compared with different models by the c statistics with 95% confidential interval (CI).

## 3 RESULT

### 3.1 Baseline characteristics

The clinical characteristics of the derivation population and validation population were shown in Table 1. For the derived data set with a total of 7806 patients, there were 253 patients diagnosed with FH using DLCN. Compared with non‐FH subjects, patients with FH were significantly younger and suffered more premature CHD (66% vs 33.8%, *P* < .001), while the prevalence of CHD was not significantly different (85.4% vs 85%, *P* = 0.874). In addition, only 12 (4.7%) patients present tendon xanthoma, which was highly specific in FH. Three patterns of statin use (high‐, moderate‐, and low‐intensity patterns) were defined on the basis of the statin type and potency as described previously.^19^ Obviously, there were much more patients with FH under high‐intensity statin treatment. In spite of moderate and high‐intensity statin treatment, patients with FH had significantly higher level of LDL‐C, TC, apoB, and Lp(a) levels than those with non‐FH (all *P* < .001). Similar results were presented in the validation population.

**Table 1:** Clinical characteristics of the derivation and validation population

	Derivation population			Validation population		
	Non‐FH	FH	*P*‐value	Non‐FH	FH	*P*‐value
Sample size	7553	253		2554	89	
Male, n (%)	5227 (69.2%)	148 (58.5%)	<.001	1676 (65.6%)	57(64.0%)	.758
Age, year	58 ± 10	50 ± 11	<.001	57 ± 11	45 ± 14	<.001
BMI, kg/(m^2^)	25.77 ± 3.26	25.81 ± 3.28	.858	26.20 ± 12.5	24.40 ± 3.48	.212
CHD, n (%)	6421 (85.0%)	216 (85.4%)	.874	1962 (78.2%)	65 (73.0%)	.244
pCHD, n (%)	2554 (33.8%)	167 (66.0%)	<.001	786 (31.4%)	53 (59.6%)	.001
Family history of CHD, n (%)	1003 (13.3%)	119 (47.8%)	<.001	260 (10.7%)	31 (35.2%)	<.001
Xanthoma, n (%)	0 (0%)	12 (4.7%)	<.001	0 (0%)	18 (20.2%)	<.001
Lipid‐lowering treatment			<.001			.001
No statin, n (%)	2671 (39.4%)	30 (12.4%)		1096 (42.9%)	26 (29.2%)	
Low potency statin, n (%)	330 (6.9%)	10 (5.8%)		89 (3.5%)	2 (2.2%)	
Medium potency statin, n (%)	1094 (23.0%)	40 (23.4%)		431 (16.9%)	10 (11.2%)	
High potency statin, n (%)	1678 (35.3%)	108 (63.2%)		938 (36.7%)	51 (57.3%)	
HT, n (%)	4593 (61.0%)	124 (51.7%)	.012	1481 (58.6%)	27 (31.4%)	<.001
DM, n (%)	2140 (28.4%)	53 (22.0%)	.080	636 (25.2%)	8 (9.3%)	.001
Smoker, n (%)	3904 (51.8%)	105 (43.8%)	.014	1242 (49.0%)	35 (39.8%)	.09
LDL‐C, mmol/L	2.51 ± 0.91	5.16 ± 2.03	<.001	2.55 ± 0.96	5.65 ± 2.35	<.001
TC, mmol/L	4.15 ± 1.06	6.96 ± 2.46	<.001	4.14 ± 1.09	7.33 ± 2.60	<.001
HDL‐C, mmol/L	1.06 ± 0.29	1.10 ± 0.34	.05	1.12 ± 0.32	1.07 ± 0.32	.101
apoB, g/L	0.92 ± 0.29	1.48 ± 0.50	<.001	0.82 ± 0.26	1.40 ± 0.45	<.001
TG, mmol/L	1.49 (1.09‐2.08)	1.71(1.29‐2.20)	<.001	1.44 (1.05‐2.02)	1.31 (0.98‐1.81)	.15
Lp(a), mg/dL	14.68 (6.55‐34.98)	28.05 (12.96‐64.79)	<.001	13.81 (6.16‐32.61)	33.48 (16.26‐60.82)	<0.001

*Note*: Data are expressed as mean ± SD, median (25th‐75th percentile) or n (%).

Abbreviations: apoB, apolipoprotein B; BMI, body mass index; CHD, coronary heart disease; DM, diabetes mellitus; FH, familial hypercholesterolemia; HDL‐C, high‐density lipoprotein cholesterol; HT, hypertension; LDL‐C, low‐density lipoprotein cholesterol; Lp(a), lipoprotein (a); pCHD, premature CHD; TC, total cholesterol: TG, triglyceride.

### 3.2 Screening criteria for FH

**Figure 1:**
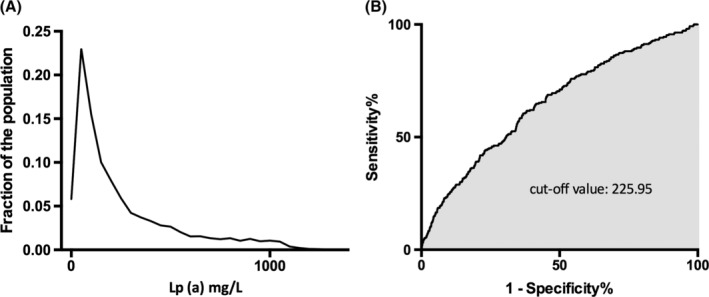
Distribution of Lp(a) levels in the derivation population (A) and its predictive value for the diagnosis of FH (B). FH, familial hypercholesterolemia; Lp(a), lipoprotein (a)

The novel modified model was shown in Table 2. The 95th percentile for LDL‐C in the untreated cohort was 4.83 mmol/L and the cut‐off value of LDL‐C for predicting FH was 4.68 mmol/L. The distributions of LDL‐C according to lipid‐lowering treatment were shown in Figure S1. Along with the DLCN criteria, examination of existing databases confirmed that Lp(a) levels ≥22 mg/dL was the best cut‐off value for a clinical diagnosis of FH (Figure 1). Thus, patients with LDL‐C < 4.7 mmol/L were given no point but patients with Lp(a) level ≥ 22 mg/dL were given one point. The DLCN criteria were also provided in Table S1.

**Table 2:** The new modified score model^a^ based on DLCN

Risk factor	Value	Points
Untreated LDL‐C	≥8.0 mmol/L	8
	6.0‐8.0 mmol/L	4
	4.8‐6.0 mmol/L	2
	<4.8 mmol/L	0
Lp(a)	≥22 mg/dL	1
	<22 mg/dL	0
Premature CHD	Yes	2
	No	0
Tendon xanthomas	Yes	6
	No	0
Family history of CHD or hypercholesterolemia	Yes	1
	No	0

Abbreviations: CHD, coronary heart disease; DLCN, Dutch Lipid Clinic Network; LDL‐C, low‐density lipoprotein cholesterol; Lp(a), lipoprotein (a).

a Total score ≥ 6 points indicate the diagnosis of FH.

### 3.3 Sensitivity/specificity analyses

We compared the distribution of FH and non‐FH patients according to DLCN and the new model as shown in Table 2. There were 91 and 52 patients were newly diagnosed with FH using the new model in the derivation and validation cohort, respectively. Agreement analysis were conducted in both the derivation population and validation population. Table 3 showed the sensitivity and specificity value of both data set. Compared to DLCN, the new modified model had 85.77% sensitivity (95% CI: 80.71%‐89.71%) and 98.79% specificity (95% CI: 98.52%‐99.02%) in the derived data set. The validation population achieved 87.64% sensitivity and 97.93% specificity. Furthermore, the new definition of FH showed a good agreement with DLCN criteria, with *κ* = 0.765 (*P* < .001) in the derived data set and 0.700 (*P* < .001) in the validated data set. In addition, the sub‐analysis of untreated patients was also conducted and revealed both good agreement with DLCN criteria in derivation and validation cohort (*κ* = 0.805 and *κ* = 0.822, respectively).

**Table 3:** Agreement between the new modified score model of FH and DLCN criteria

	Derivation population		Validation population	
	All cohort	Untreated cohort	All cohort	Untreated cohort
Sensitivity, % (95% CI)	85.77 (80.71‐89.71)	76.67 (57.30‐89.37)	87.64 (78.55‐93.37)	73.08 (51.95‐87.65)
Specificity, % (95% CI)	98.79 (98.52‐99.02)	98.85 (98.59‐99.95)	97.93 (97.27‐98.43)	99.91 (99.39‐99.99)
Positive predictive value, % (95% CI)	70.45 (64.96‐75.43)	85.19 (65.39‐95.14)	0.60 (51.02‐68.38)	95.00 (73.06‐99.74)
Negative predictive value, % (95% CI)	99.52 (99.33‐99.66)	99.74 (99.44‐99.89)	99.55 (99.18‐99.76)	99.35 (98.6‐99.71)
Ƙ coefficient	0.765	0.805	0.700	0.822
*P*‐value	<.001	<.001	<.001	<.001

Abbreviations: CI, confidence interval; DLCN, Dutch Lipid Clinic Network; FH, familial hypercholesterolemia.

Furthermore, we compared the agreements between DLCN criteria, new model and the genetic analysis as shown in Table S3. The results showed that the new model had a little better agreement with the mutation than DLCN, but both of them showed poor agreement with mutations (*κ* = 0.355 vs *κ* = 0.340). of note, the new model had a moderate agreement with mutations in the validation cohort (*κ* = 0.470).

### 3.4 Predictive performance for FH

**Figure 2:**
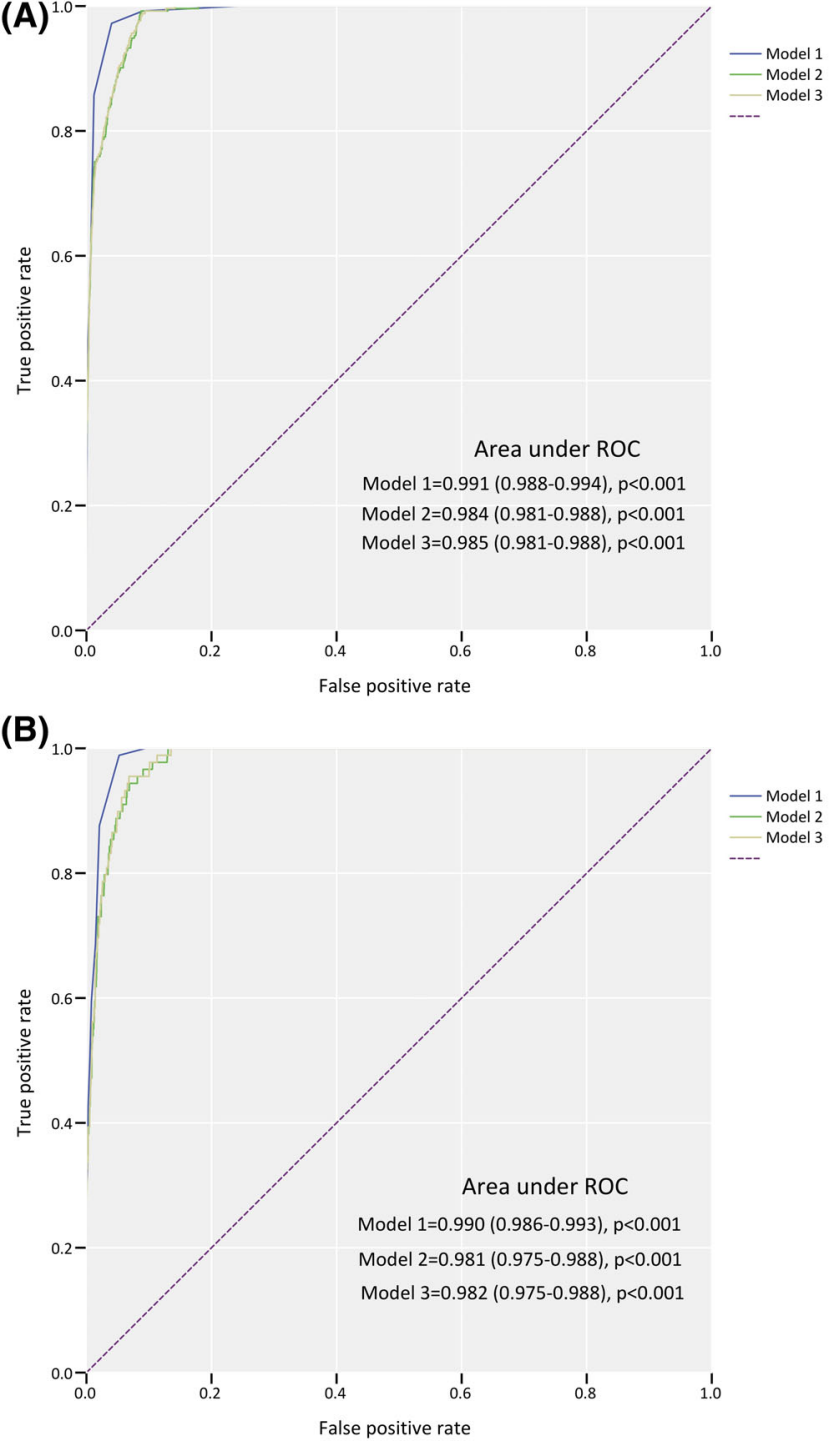
Receiver‐operating characteristic curve of different models in derived data set (A) and validated data set (B). Model 1: new modified model; Model 2: untreated LDL‐C level; Model 3: untreated LDL‐C level + Lp (a) level. LDL‐C: low‐density lipoprotein cholesterol; Lp (a): lipoprotein (a); ROC: receiver‐operating characteristic curve

The predictive capacity of the new modified model was evaluated using c statistic (Figure 2). The new modified model has shown high discrimination (area under curve [AUC] 0.991, 95% CI: 0.988‐0.994, *P* < .001) for distinguishing clinical FH from non‐FH diagnosed using DLCN, which was higher than sole LDL‐C level and LDL‐C plus Lp(a) level. External validation also indicated an excellent discriminatory power (AUC 0.990, 95% CI: 0.986‐0.993).

## 4 DISCUSSION

In the current study, we have firstly established novel diagnostic criteria for FH including Lp(a) as an index. The new definition for FH was modified from DLCN criteria and relied on (a) LDL‐C level; (b) Lp(a) level; (c) prevalence of premature CHD; (d) presence of tendon xanthomas; (e) family history of CHD and/or hypercholesterolemia in first‐degree relatives. This new modified score model showed good discrimination for recognizing clinical FH and high degree of agreement with the widely used DLCN criteria, indicating the change of LDL‐C stratification and addition of Lp(a) still brought the novel model powerful diagnostic performance.

Worldwide, there is no “gold standard” for diagnosis of FH and diagnostic algorithms varies across countries. In China, there has been no widely accepted definition of FH in the clinical practice until now. Among the diagnostic criteria, DLCN and SBR are the most used but with several limitations. We modified the DLCN criteria and established this new model. Except for including Lp(a), the modifications mainly focused on LDL‐C cut‐off value and physical signs. First, the LDL‐C cut‐points of DLCN criteria has been changed because the date deriving from western populations may not be adopted to Chinese population due to the relatively lower level of LDL‐C in China.^23^ The FH diagnostic guideline issued by Japan applies LDL‐C ≥ 180 mg/dL (4.6 mmol/L) as the cut‐point.^24^ Korean scientists found that best LDL‐C threshold for predicting putative mutation was 225 mg/dL (5.8 mmol/L) while the conventional diagnostic criteria had low specificities.^25^ Thus, we downgraded the LDL‐C cut‐points based on the Chinese data. Second, the physical signs including xanthomas and corneal arcus are uncommon despite of high specificity.^26^ Also, the signs often rely on visual determination subjectively by physicians and could sometimes bring confusions because of obscurity. Furthermore, not all the lipid deposition in connective tissue of the skin, tendons or fasciae are the results of FH.^27^, ^28^ The differential diagnosis of xanthomas includes sitosterolemia and cerebrotendinous xanthomas.^29^, ^30^ However, considering the high specificity of tendon xanthomas, relatively prevalent among homozygous FH especially, we still kept it in the new model with six points.

We firstly try to introduce Lp(a) level as one indicator in the diagnosis criteria of FH. Lp(a) is LDL‐like particle composed of apolipoprotein B100 but with distinctive physiological effect.^31^ Previous studies have demonstrated the significantly elevated level of Lp(a) in patients with FH.^32^ Furthermore, high level of Lp(a) can inflate LDL measurements and could explain the phenotypes of parts of patients with FH, especially those without detected pathological mutations.^33^ The Danish study conducted by Langsted et al found that high Lp(a) level and *LPA* risk genotypes may account for a quarter of individuals diagnosed with clinical FH.^34^ In addition, recent publications have found that high level of Lp(a) was an important predictive variable for CHD risk in patients with FH.^35^, ^36^ Furthermore, increasing applications of PCSK9 inhibitors and apheresis in the clinical practice have made it possible to decrease Lp(a) levels.^37^ Thus, we should pay more attentions to Lp(a) and the biochemical measurement of Lp(a) is recommended for a patient with suspected FH. In the current study, we highlighted the importance of Lp(a) and assigned one point for patients with Lp(a) ≥ 22 mg/dL, concerning on those without extremely high level of LDL‐C but with high level of Lp(a) and increased risk of CHD.

The severe cardiovascular complications are the main driving force for early diagnosis and treatment of FH. Timely and definite diagnosis of FH could help to start decreasing the risk at a young age and extend across the lifespan. Even though the new modified score model may be arbitrary and limited, the integration of Lp(a) could provide a novel sight into the diagnosis of FH and assist identifying patients who carry a high risk of cardiovascular disease. Moreover, the statistical analysis has proven its good agreement with the current used DLCN criteria. Of course, the comparison between the new model and genetic analysis did not show a good agreement between them. However, the results were not surprising. First, the possible explanation may be the heterogeneity between phenotype and genotype. Second, as we all know, Lp(a) is determined by *LPA* gene and the genetic analysis for FH covering *LDLR, APOB*, and *PCSK9* genes would not show a large improvement in regard of the agreement. After all, there is no gold standard for FH diagnosis.

We acknowledge the limitations in the current study. First, the new score model was modified mainly according to our experiences in the clinical practice and supported by our own data. Further analysis to establish a more scientific model is essential. Second, the DLCN criteria are not applicable for children. So is the new modified model.

## 5 CONCLUSION

We first proposed the integration of Lp(a) as an indicator into the diagnosis of FH and modified the DLCN criteria based on data of Chinese population. The novel modified model highlighted the importance of Lp(a) and could provide new insights into FH diagnosis. With special adaption in Chinese population, this model is expected to facilitate diagnosis and, most importantly, help prevent cardiovascular complications.

## CONFLICT OF INTEREST

The authors declare no potential conflict of interests.

## Supporting information


**TABLE S1** Dutch Lipid Clinic Network criteria for diagnosis of familial hypercholesterolemia
**TABLE S2.** The distribution of patients according to Dutch Lipid Clinic Network and new model
**TABLE S3** Agreement between the new modified score model of familial hypercholesterolemia, Dutch Lipid Clinic Network criteria and mutation analysis
**FIGURE 1** Distribution of the low‐density lipoprotein cholesterol (LDL‐C) levels in the derivation population. (A) population without lipid‐lowering treatment (n = 3482); (B) population with lipid‐lowering treatment (n = 4324). LDL‐C: low‐density lipoprotein cholesterol
