# Supplementary material for: A modified algorithm with lipoprotein(a) added for diagnosis of familial hypercholesterolemia
Source: Clin Cardiol. 2019 Aug 22;42(10):988–94. doi: 10.1002/clc.23251 (PMC6788465; doi:10.1002/clc.23251)

**SUPPLEMENTAL MATERIAL**

## Development and validation of a modified diagnostic algorithm including lipoprotein(a) for familial hypercholesterolemia

## Di Sun^1#^, Ye-Xuan Cao^1#^, Sha Li^1^, Yuan-Lin Guo^1^, Na-Qiong Wu^1^, Cheng-Gang Zhu^1^, Ying Gao^1^, Qiu-Ting Dong^1^, Geng Liu^1^, Qian Dong^1^, Jian-Jun Li^1*^

## ^#^ The first two authors contributed equally.

**Supplemental Table 1.** DLCN criteria for diagnosis of FH.

| Risk factor | Value | Point |
| --- | --- | --- |
| Family history | First-degree relative with known premature CHD; OR | 1 |
|  | First-degree relative with known LDL-C>95th percentile by age and gender for country; OR | 1 |
|  | First-degree relative with tendon xanthoma and/or corneal arcus; OR | 2 |
|  | Child(ren) ,18 years with LDL-C >95th percentile by age and gender for country | 2 |
| Personal history | premature CHD | 2 |
|  | premature cerebral or peripheral vascular disease | 1 |
| Physical examination | Tendon xanthoma | 6 |
|  | Corneal arcus in a person <45 years | 4 |
| LDL-C | >8.5 mmol/L | 8 |
|  | 6.5–8.4 mmol/L | 5 |
|  | 5.0–6.4 mmol/L | 3 |
|  | 4.0–4.9 mmol/L | 1 |
| DNA analysis | Causative mutation shown in the LDLR, APOB, or PCSK9 genes | 8 |

*Total scores > 8 points indicate a ‘definite FH’ and scores 6 to 8 make a ‘probable FH’.

DLCN: Dutch Lipid Clinic Network; FH: familial hypercholesterolemia; CHD: coronary heart disease; LDL-C: low-density lipoprotein cholesterol

**Supplemental Table 2.** The distribution of patients according to DLCN and new model.

|  | derivation cohort | |  | validation cohort | |
| --- | --- | --- | --- | --- | --- |
|  | new model-nonFH | new model-FH |  | new model-nonFH | new model-FH |
| DLCN-nonFH | 7462(99.5%) | 91(29.5%) |  | 2454(99.6%) | 52(40%) |
| DLCN-FH | 36(0.5%) | 217(70.5%) |  | 11(0.4%) | 78(60%) |

FH: familial hypercholesterolemia; DLCN: Dutch Lipid Clinic Network;

**Supplemental Table 3.** Agreement between the new modified score model of FH, DLCN criteria and mutation analysis.

|  | derivation (n=530) | |  | validation (n=188) | |  | overall (n=718) | |
| --- | --- | --- | --- | --- | --- | --- | --- | --- |
|  | DLDN-FH | new model-FH |  | DLDN-FH | new model-FH |  | DLDN-FH | new model-FH |
| Mutation (+) | 105 (59%) | 106 (59.6%) |  | 67 (67.7%) | 69 (69.7%) |  | 172 (62.1%) | 177 (63.9%) |
| Mutation (-) | 102 (29%) | 103 (29.3%) |  | 20 (22.5%) | 20 (22.5%) |  | 122 (27.7%) | 123 (27.9%) |
| Ƙ coefficient | 0.288 | 0.29 |  | 0.449 | 0.47 |  | 0.34 | 0.355 |

FH: familial hypercholesterolemia; DLCN: Dutch Lipid Clinic Network;

**Supplemental Figure 1. Distribution of the LDL-C levels in the derivation population. (A) population without lipid-lowering treatment (n=3482); (B) population with lipid-lowering treatment (n=4324).** LDL-C: low-density lipoprotein cholesterol


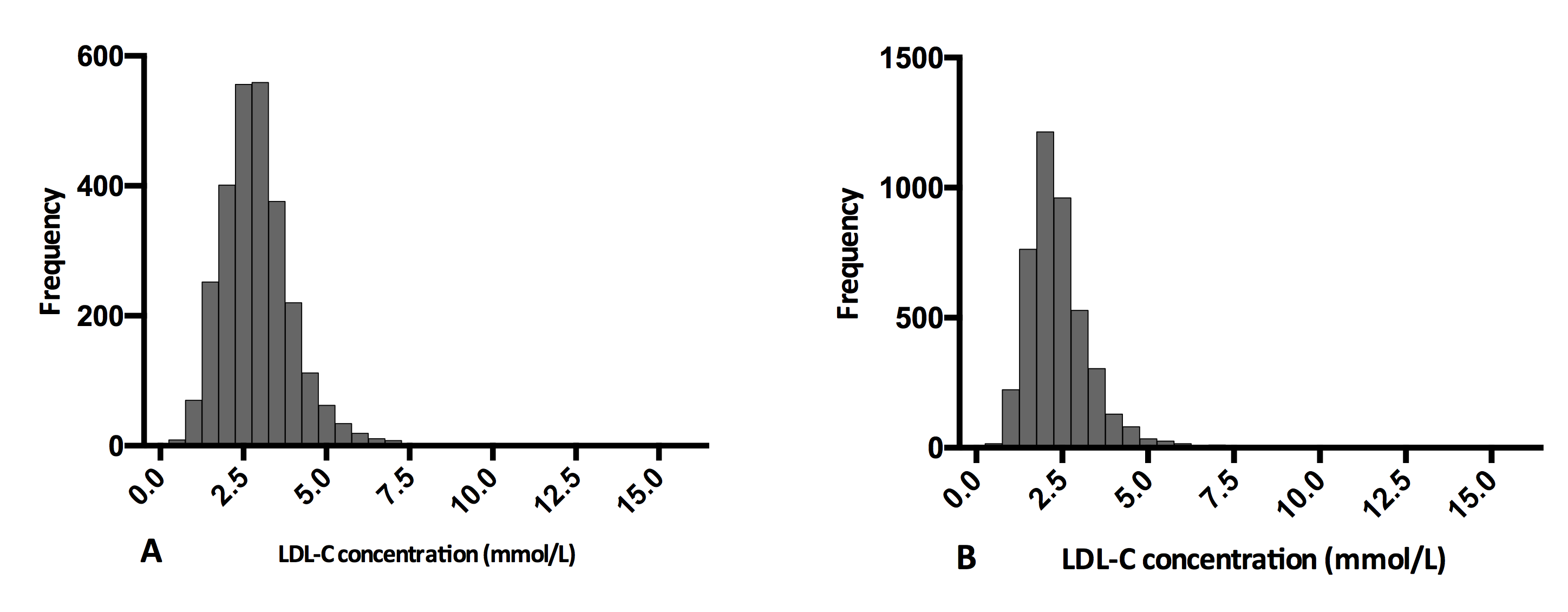

Supplement: Supplementary file 1 — TABLE S1 Dutch Lipid Clinic Network criteria for diagnosis of familial hypercholesterolemia TABLE S2. The distribution of patients according to Dutch Lipid Clinic Network and new model TABLE S3 Agreement between the new modified score model of familial hypercholesterolemia, Dutch Lipid Clinic Network criteria and mutation analysis FIGURE 1 Distribution of the low‐density lipoprotein cholesterol (LDL‐C) levels in the derivation population. (A) population without lipid‐lowering treatment (n = 3482); (B) population with lipid‐lowering treatment (n = 4324). LDL‐C: low‐density lipoprotein cholesterol [file CLC-42-988-s001.docx]
